# Dehydroxylate I formation from the thermal decomposition of serpentine on c-complex asteroids: similarities to carlosturanite

**DOI:** 10.1186/s40623-025-02310-w

**Published:** 2025-12-09

**Authors:** Laura E. Jenkins, Ashley J. King, Martin R. Lee, Luke Daly, Konstantin Ignatyev, Cameron J. Floyd, Pierre-Etienne M. C. Martin

**Affiliations:** 1 School of Geographical and Earth Sciences, The University of Glasgow, Glasgow, Scotland, UK G12 8RZ; 2 Planetary Materials Group, Natural History Museum, London, England, UK SW7 5BD; 3 Space Science and Technology Centre, School of Earth and Planetary Sciences, Curtin University, Perth, 6845 Australia; 4 Australian Centre for Microscopy and Microanalysis, The University of Sydney, Sydney, 2006 Australia; 5 Diamond Light Source, Harwell Science and Innovation Campus, Didcot, Oxfordshire OX11 0DE England, UK

**Keywords:** Serpentine, Carbonaceous chondrites, Meteorites, Thermal metamorphism, Post-hydration heating, X-ray diffraction, Laboratory experiments

## Abstract

Dehydroxylate I, a product of the thermal decomposition of serpentine, has been observed in heated carbonaceous chondrite meteorites. To better understand the occurrence of dehydroxylate I on carbonaceous asteroids, we have experimentally heated the carbonaceous chondrite Murchison from 400 to 550 °C at 25°C temperature steps, during which in situ micro X-ray diffraction (µXRD) patterns were collected using synchrotron radiation. µXRD was utilized such that the dehydroxylate I’s diffraction pattern could be isolated and characterized. This was successfully achieved, with the phase being detected at 400 °C. A diffraction pattern for dehydroxylate I was isolated at 525 °C, where it displayed crystallographic similarities to the mineral carlosturanite. We propose dehydroxylate I is produced when gaps form in serpentine’s tetrahedral sheet during its breakdown, which is consistent with previous studies on serpentine decomposition. The d-spacings for dehydroxylate I described here can be used to better identify it in natural and experimentally heated terrestrial and meteoritic samples.

**Graphical Abstract:**

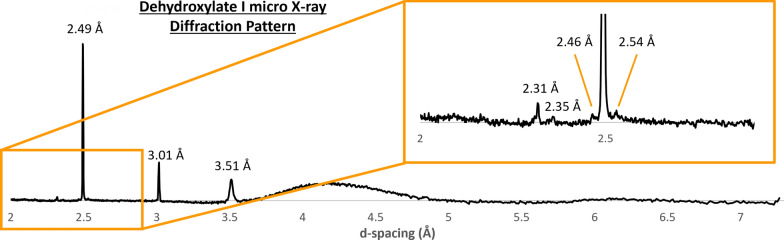

## Introduction

C-complex asteroids, the parent bodies of aqueously altered carbonaceous chondrite meteorites, are important volatile reservoirs in the solar system and may have contributed water to the early Earth (Alexander et al. 2012). These aqueously altered carbonaceous chondrites often contain serpentine ((Mg,Fe)_3_Si_2_O_5_(OH)_4_), which incorporates hydroxyl into its crystal structure (Howard et al. 2011; Rubin et al. 2007). However, some of these serpentine-bearing carbonaceous chondrites have been heated after aqueous alteration in a process known as post-hydration heating, which alters their parent asteroids’ volatile contents. Many, but not all, of these aqueously altered and subsequently heated meteorites are Mighei-like carbonaceous (CM) chondrites (Nakamura 2005), which often have serpentine as their most abundant mineral (Howard et al. 2011). The cause(s) of post-hydration heating have yet to be constrained, so it is important to understand how it progresses as it will have implications on which processes could be responsible for it.

Post-hydration heating occurs at a wide range of temperatures, from ~ 150–1000 °C, for short durations, from hours to weeks (King et al. 2019; Nakamura 2005; Nakato et al. 2008). Due to its variability in peak temperature, a plethora of different effects can arise from post-hydration heating. Volatile bearing minerals, like tochilinite (2(Fe,Ni,Cu)_1-x_S · n(Mg,Fe)(OH)_2_; *x* = 0.09–0.29, *n* = 1.58–1.85; Palmer and Lauretta 2011) and serpentine, break down, and from these decomposed remnants, secondary anhydrous minerals (e.g., troilite (FeS), olivine ((Mg,Fe)SiO_4_)) form (Nakamura 2005; Jenkins et al. 2024). Serpentine itself begins to break down at 300 °C, recrystallizing into olivine and occasionally pyroxene ((Mg,Fe)_2_Si_2_O_6_) at temperatures above 500 °C (Akai 1992; Nakamura 2005; Jenkins et al. 2024).

In our previous investigation of the progression of mineral transitions during post-hydration heating, we experimentally heated two CM chondrite meteorites, Allan Hills (ALH) 83100 and Murchison, while simultaneously collecting bulk powder X-ray diffraction (pXRD) data (Jenkins et al. 2024). From these pXRD patterns, we observed a phase that appeared at 525 °C and increased in abundance until 750 °C was reached, wherein it broke down. This short-lived phase is represented by only three peaks (d=3.53, 3.56, and 3.67 Å), in part likely due to peak overlap with the numerous minerals constituting ALH 83100 and Murchison, making its identification challenging. Even the peaks we did observe commonly overlapped with enstatite, such that their identification was only achievable due to those peaks beginning to weaken at 750 °C despite enstatite being stable at that temperature.

Due to this phase’s appearance after serpentine began to break down, at 300 °C, the two reactions were interpreted to be closely related (Jenkins et al. 2024). This was not the first report of such a phase resulting from serpentine decomposition. Akai (1988) studied three naturally heated CM chondrites with transmission electron microscopy and found that their matrices were composed of a disordered phase that shared aspects with both serpentine and olivine. Akai (1988; 1992) was able to recreate this phase by experimentally heating Murchison at 400–600 °C, reporting that it is a transitional phase that occurs during serpentine decomposition, prior to its recrystallization into olivine and pyroxene.

This transitional phase occurring in heated CM chondrite meteorites was proposed to be dehydroxylate I by Jenkins et al. (2024). Dehydroxylate I is one of two transitional phases resulting from the thermal decomposition of serpentine, the other being dehydroxylate II (MacKenzie and Meinhold 1994). As heating progresses, dehydroxylate I eventually recrystallizes into olivine, while dehydroxylate II transforms into pyroxene at higher temperatures (MacKenzie and Meinhold 1994).

Several studies have investigated the thermal decomposition of serpentine and chrysotile and how it progresses in relation to these phases, with three models being developed (Ball and Taylor 1963; Brindley and Hayami 1965; MacKenzie and Meinhold 1994). However, there is still much unknown about the structures of dehydroxylate I and II, and how they transform into anhydrous silicates. A monomineralic X-ray diffraction pattern, bulk or in situ micro*,* has yet to be produced for dehydroxylate I or II; the d-spacings for dehydroxylate I and II’s X-ray diffraction peaks have also yet to be identified. Additionally, much of this previous work focused on monomineralic chrysotile (e.g., Brindley and Hayami 1965; MacKenzie and Meinhold 1994); monoclinic chrysotile has a different crystal structure to triclinic serpentine, while monomineralic serpentine is known to decompose at a different rate than serpentine in polymineralic samples like CM chondrites, which are composed of not only different varieties of serpentine (lizardite, cronstedtite, and intermediate compositions), but of several other minerals (Velbel and Zolensky 2021). To ensure that experimental results on terrestrial serpentine and chrysotile are applicable to CM chondrites, it is important to study polymineralic samples.

Herein, we focus on the formation of dehydroxylate I in heated CM chondrite meteorites by serpentine decomposition. We experimentally heated two rock slices of the CM chondrite Murchison to 400–550 °C and used X-ray diffraction on the I18 beamline at Diamond Light Source (DLS) to characterize dehydroxylate I. To minimize the chance of peak overlap occurring, we collected in situ micro X-ray diffraction (µXRD) patterns during heating using synchrotron radiation. µXRD involves the analysis of unpowdered samples (e.g., rock slices). Orientation plays a strong role, with µXRD patterns showing a portion of the peaks that would normally be displayed in a pXRD pattern and peak intensities varying depending on orientation. However, µXRD is non-destructive, allowing for analysis of rare samples (Flemming 2007). With µXRD, it is possible to target specific areas, allowing minerals to be studied in their original context and minimizing peak overlap with other minerals that may be present in the bulk rock. This was our approach to investigating serpentine dehydration within the Murchison meteorite, allowing dehydroxylate I to be isolated and characterized.

## Materials and methods

Murchison is a CM2 chondrite that is composed of ~ 50 vol% cronstedtite and tochilinite, ~ 22 vol% lizardite, ~ 15 vol% olivine, ~ 8 vol% orthopyroxene, ~ 2 vol% sulphides, ~ 1 vol% calcite, and ~ 1 vol% magnetite (Howard et al. 2011); it has a petrologic subtype of 2.5 (Rubin et al. 2007). Two polished rock slices of Murchison obtained from the Natural History Museum (NHM), London were used for this experiment. Both rock slices are 1.8 mm^2^ in area and ~ 100 µm thick. These rock slices shall hereby be referred to as M_A and M_B.

**Fig. 1:**
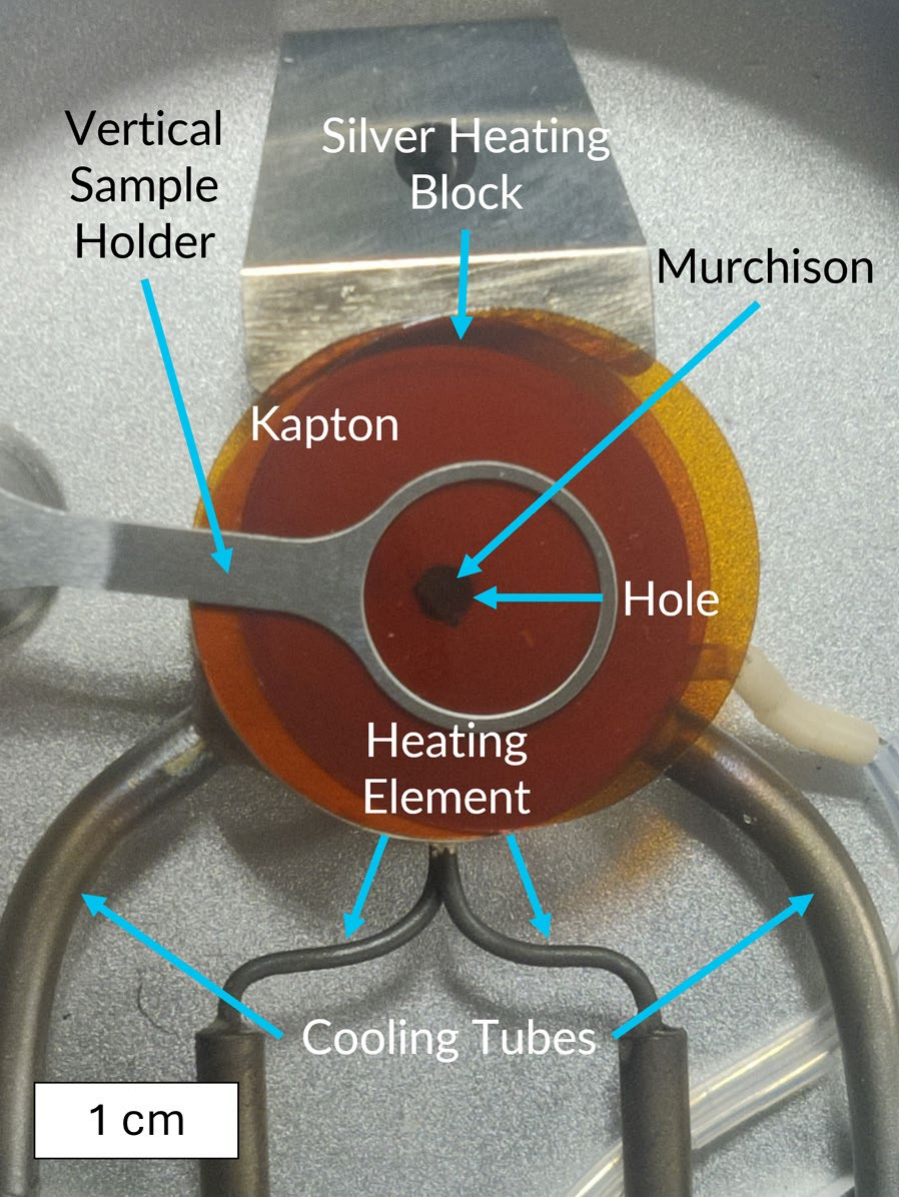
Sample setup inside the THMS600 Linkam cell. A polished rock slice of Murchison is held in place between two Kapton disks by a vertical sample holder

The samples were experimentally heated in a THMS600 Linkam cell equipped with Kapton windows and connected to a TMS 94 temperature controller and an ECP water circulation pump. The Linkam cell had to be positioned vertically, and a vertical sample holder was installed in the Linkam cell to ensure M_A and M_B were held in place throughout the experiment. The THMS600 Linkam cell’s vertical sample holder is designed to hold the sample in place between two disks by friction atop the silver heating block designed to conduct heat to the sample (Fig. 1). The disks used in this experiment were Kapton, which was chosen for its X-ray transparency, as well as being sufficiently flexible not to crush the samples. To ensure the samples were heated in a non-oxidizing environment, N_2_ gas was continuously pumped through the Linkam cell.

µXRD patterns were collected in transmission on the I18 beamline at DLS. The I18 beamline is equipped with an in-vacuum 27 mm undulator, a Si (111) double crystal monochromator, and three sets of focusing mirrors. An Excalibur X-ray diffraction detector was used to collect µXRD patterns for an array of targets covering areas of 7.73 × 10^–3^ mm^2^ (528 targets) and 3.23 × 10^–2^ mm^2^ (306 targets) for M_A and M_B, respectively. An X-ray wavelength of 0.826 Å and a spot size of 2 μm were used. For more information regarding beamline specifications, see Mosselmans et al. (2009).

Prior to heating, µXRD patterns for the areas in each sample were collected at room temperature. The sample was then heated to the first temperature step of 400 °C at a rate of 12° per minute. It was held at temperature for two hours, with µXRD patterns collected for the same area after the first hour. After two hours had passed, the sample was heated by 25 °C to the next temperature step (e.g., 425 °C), wherein the process was repeated. Temperature steps and timescales were designed to follow as closely as possible to that of our previous pXRD experiment on the I11 beamline at DLS (see Jenkins et al. 2024), wherein heating timescales of hours were used to ensure both that the temperature would equilibrate throughout the sample and that mineral reactions could have enough time to occur.

The original aim of the experiment was to heat the samples from 400 to 600 °C with the samples fixed in place in such a manner that crystallographic changes in specific areas could be tracked. However, Kapton has a melting point of 400 °C (Ahmed et al. 2012), which was not known when the experiments were designed. Upon heating, Kapton disks affixing the samples in place changed enough to cause samples to shift slightly and at 550 °C, the Kapton disintegrated entirely, causing the sample to fall from the Linkam cell. μXRD patterns were collected up to 550 °C, however, due to the samples shifting, no singular target (i.e. a specific point within the rock slice) could be tracked. As a water cooler was used, the Kapton windows on the Linkam cell were not heated to 400 °C and were unaffected. To assess the effect of melting Kapton on the µXRD patterns, a pair of Kapton disks were heated without any samples at the same temperature-timescales (400–550 °C at 25°C steps, spending two hours at each step), during which, at the same increments (after an hour at each step), µXRD patterns were collected.

To assist with mineral identification, µXRD patterns for several powdered standards were collected. The standards were also affixed between two pieces of Kapton; µXRD patterns for these standards were acquired at room temperature. The standards were pyrrhotite (BM1924,1316), cronstedtite (BM52294), magnetite (BM57974), enstatite (BM1933,407), antigorite (BM66586), pentlandite (BM1975,466), lizardite (Lizard Peninsula), olivine (San Carlos), calcite (Iceland spar), augite (Bohemia), and chrysotile (BM1929,1974). Identification of troilite was done using the d-spacings of peaks from the Crystallography Open Database (COD) card #9004036. All µXRD patterns collected in this study were processed with both Data Analysis WorkbeNch (DAWN) 2.27 and Rigaku SmartLab II software. For more information regarding DAWN, please see Basham et al. (2015).

## Results

**Fig. 2:**
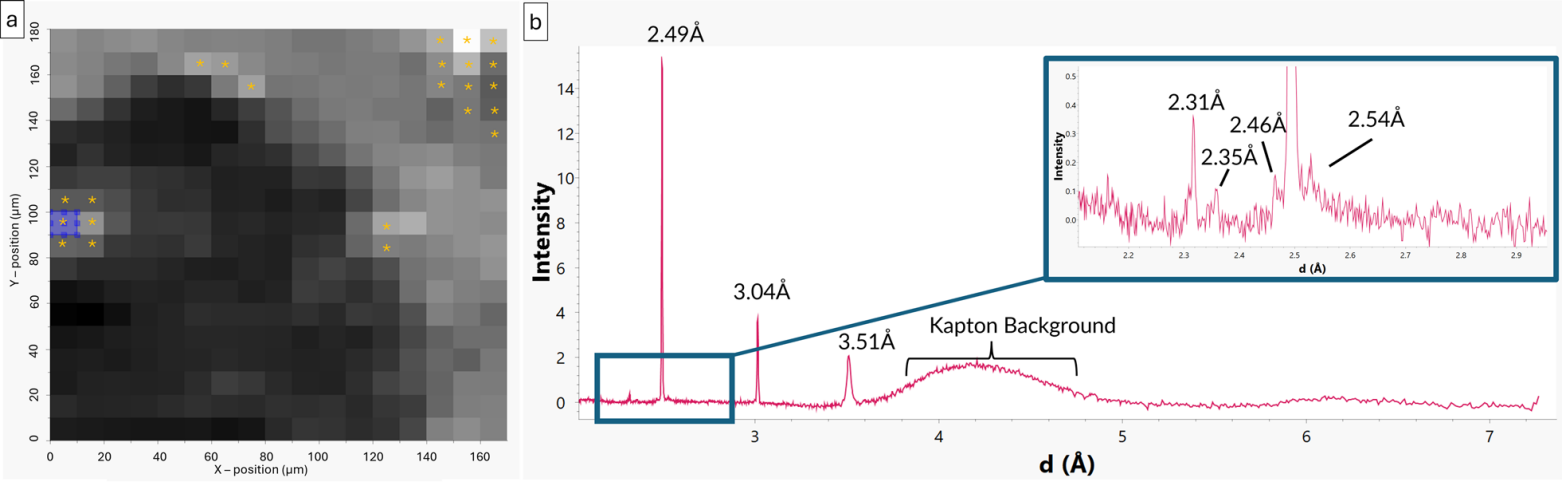
M_B at 525 °C. **a** Grid of targets from where the XRD data were collected, with the shade of grey corresponding to the intensity of diffraction at *d* = 3.5 Å. Each target had its μXRD pattern collected using a spot size of 2 μm. Lighter shades of grey indicate a higher diffraction intensity. Some areas have larger intensities due to melting Kapton adding background noise. Locations where dehydroxylate I was detected are marked with gold stars. A blue box marks out the target from where the XRD pattern in **b** is taken. **b** XRD pattern showing exclusively dehydroxylate I. d-spacings of peaks are labelled

A grid of diffraction targets was taken at each temperature step, which can be presented in greyscale with the shade of grey corresponding to the intensity of diffraction at a predefined d-spacing; whiter squares correspond to a higher intensity (Fig. 2a). Patterns for individual targets can be viewed (Fig. 2b) and all diffraction patterns for the area can be summed into a single “bulk” pattern. These data were both used to assess overall mineral changes within the sample as well as analyse dehydroxylate I.

### The effects of Kapton melting

**Fig. 3:**
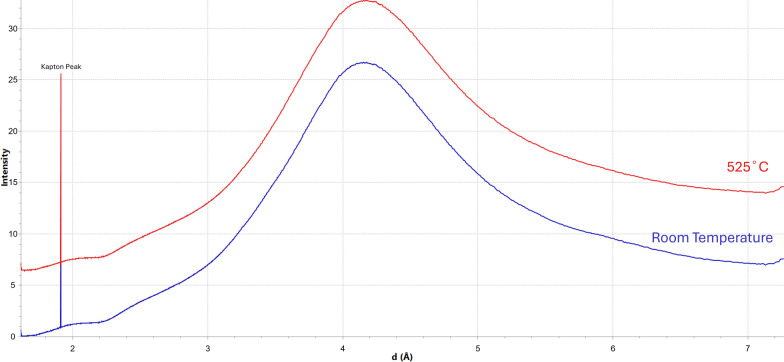
Comparison of XRD patterns for Kapton at room temperature (blue) and 525 °C (red). No significant differences can be seen. The Kapton soon disintegrated at 550 °C

The heating of Kapton blanks had little effect on the diffraction patterns. The heated Kapton added intensity to the pattern, with some areas experiencing substantially higher background, but no additional peaks appeared in the patterns (Fig. 3). However, melting of Kapton has led to considerable sample shifting in the M_A experiment, which has resulted in a reduced sample area for analysis.

### Mineral transitions during heating

**Fig. 4:**
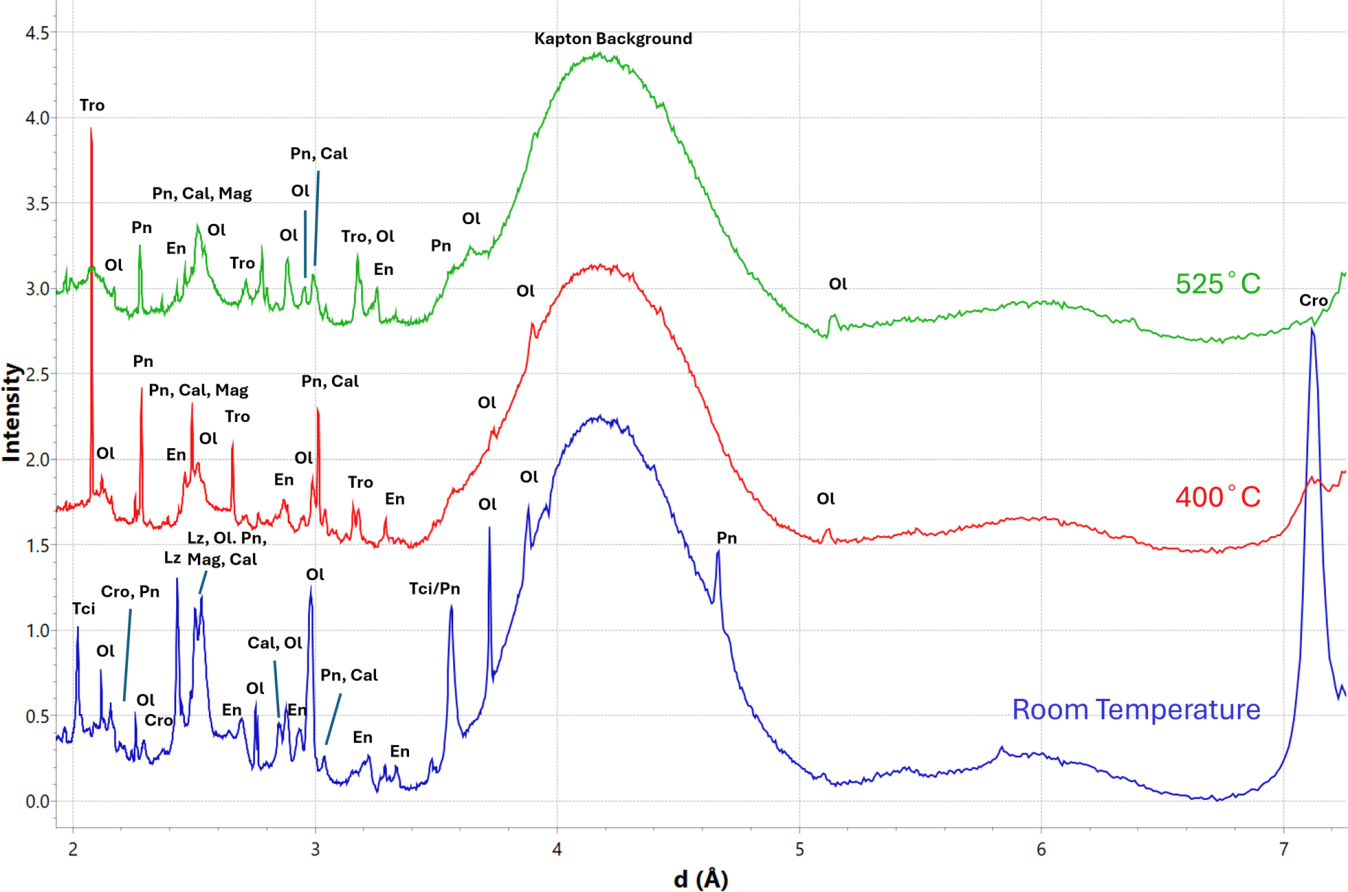
Comparison of summed XRD patterns for M_A at room temperature (blue),at 400 °C (red), and at 525 °C (green). Tochilinite (Tci), olivine (Ol), pentlandite (Pn), cronstedtite (Cro), lizardite (Lz), enstatite (En), calcite (Cal), magnetite (Mag), and troilite (Tro) are labelled. Cro, Lz, and Tci are present in the room temperature pattern, but are largely absent at 400 °C. Tro is not present at room temperature but appears at 400 °C

**Fig. 5:**
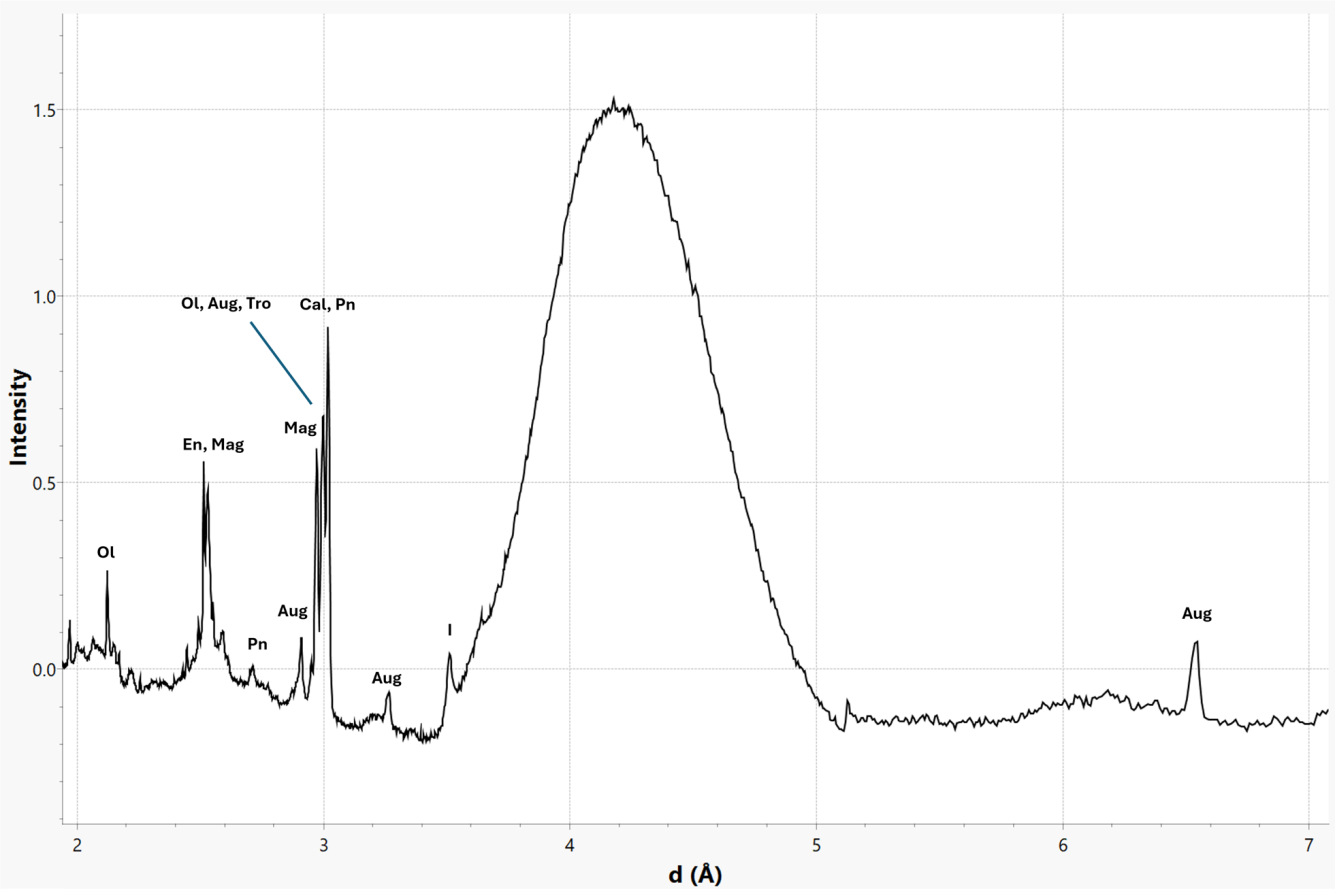
Bulk μXRD pattern of M_B at 525 °C showing major phases present. These phases include olivine (Ol), enstatite (En), magnetite (Mag), pentlandite (Pn), augite (Aug), troilite (Tro), calcite (Cal), and dehydroxylate I (I)

When M_A and M_B were heated, peaks associated with various phases often changed due to the samples moving; individual olivine peaks would disappear and reappear as olivine grains of different orientations entered and exited the data collection area. Mineral transitions were tracked by using the pattern for the summed area to ensure a peak disappearance and appearance was not due to sample movement. Initially, M_A and M_B at room temperature were composed of tochilinite, olivine, lizardite, cronstedtite, enstatite, calcite, magnetite, and pentlandite. In M_A, at 400 °C, all peaks associated with tochilinite and lizardite disappeared, coinciding with the appearance of troilite peaks. Additionally, most cronstedtite peaks also disappeared, with only its most intense peak at 7.13 Å being visible in the patterns (Fig. 4). This final cronstedtite peak disappeared at 525 °C. In M_B, all tochilinite, lizardite, and cronstedtite peaks disappeared at 400 °C and, like with M_A, troilite peaks also appeared at that same temperature. In M_B, at 500 °C, peaks associated with augite appear, however peaks associated with calcite are still present up until the experiment’s completion (Fig. 5).

### Isolation of dehydroxylate I

To identify dehydroxylate I, the grid of diffraction targets was set for their shade of grey to correspond to the intensity of diffraction at *d* =  ~ 3.5 Å (Fig. 2a), the peak at which dehydroxylate I was most commonly identified by Jenkins et al. (2024). Each target that had a high diffraction intensity at ~ 3.5 Å was analysed for dehydroxylate I.

**Fig. 6:**
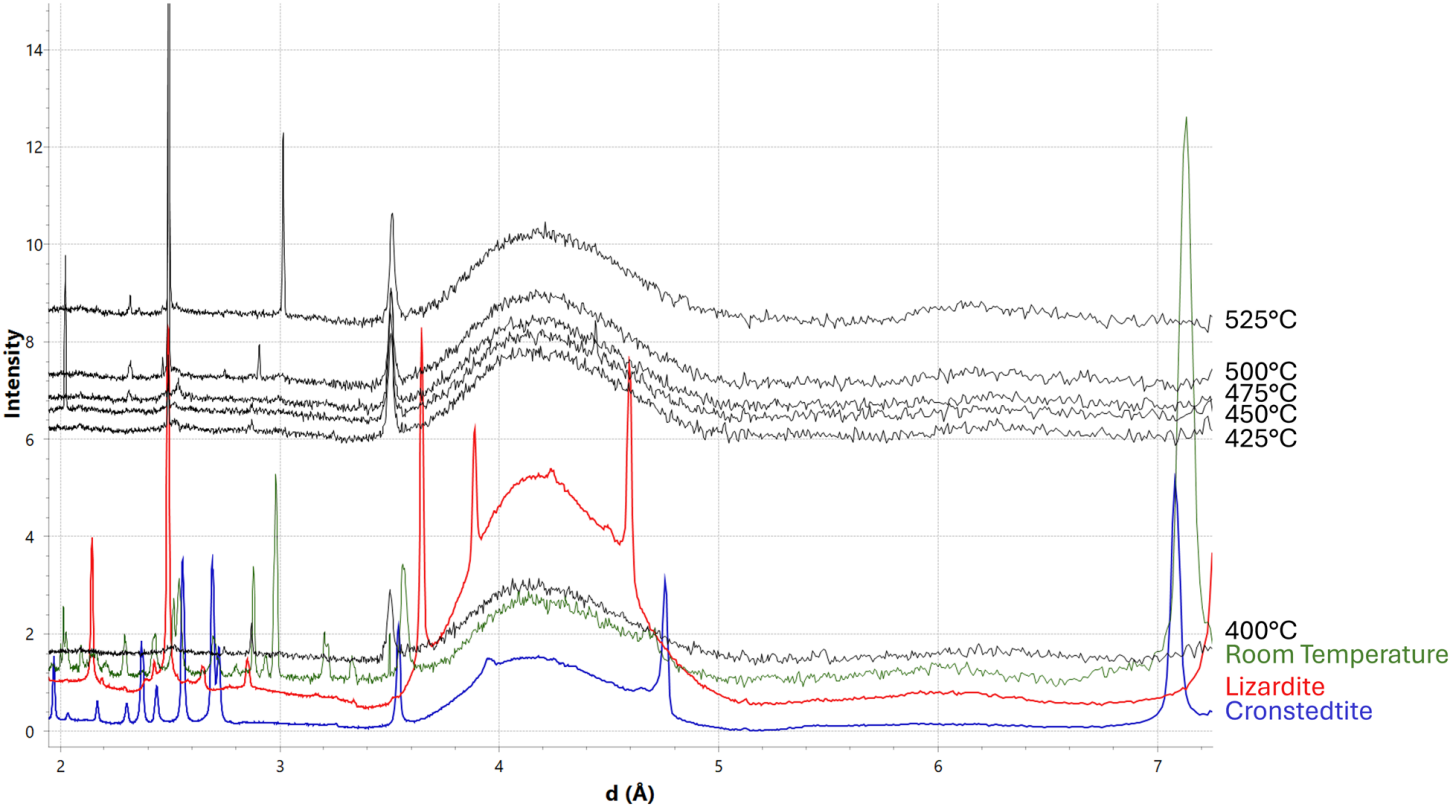
μXRD patterns from select targets in M_B (black and green) showing the evolution of serpentine throughout heating compared to lizardite (red) and cronstedtite (blue) standards. The 3.56 Å serpentine peak shifts to 3.51 Å at 400 °C as other serpentine peaks decrease in intensity. At 425 °C, there is a large increase in background, likely due to the Kapton melting

The 3.51 Å peak was first observed at 400 °C, however was most prevalent at 525 °C. The production of the 3.51 Å peak appears to be due to serpentine peaks at 3.56 Å shifting (Fig. 6). In M_A, at 525 °C, three targets that contain a peak at ~ 3.5 Å were found, however, these three targets also contained peaks that matched enstatite, olivine, and pentlandite. The targets may have been subject to peak overlap by enstatite, olivine, and pentlandite. The lack of targets in M_A is due to the sample shifting during the melting of Kapton. In M_B, at 525 °C, 23 targets were found to contain a peak at ~ 3.5 Å (Fig. 2a). Twenty of the targets had peaks that corresponded to other minerals (e.g., enstatite, olivine, magnetite, calcite), while two targets had only the 3.51 Å peak. However, one target had peaks other than the 3.51 Å peak that did not match any other phase (Fig. 2) and thus was not affected by peak overlap. These peaks are all attributed to the dehydroxylate I. No matches were found with the standards used.

## Discussion

Despite Kapton melting during the experiment, dehydroxylate I was identified and isolated in the heated Murchison samples. In addition to characterizing dehydroxylate I, here we describe how Kapton melting affected the environment the Murchison samples were heated in and how it affected data, as well as how the mineral reactions observed compare to previous studies.

### Melting Kapton and the environment experienced by the heated samples

The Kapton blanks that were heated at the same temperature-timescales as the M_A and M_B showed little change. There was added intensity to the background as heating progressed, however no additional peaks were visible in the patterns. The melting Kapton had minimal effect on the data in regard to the diffraction peaks observed.

Regarding the environment the samples were heated in and how the Kapton may have affected it, N_2_ was continuously pumped through the Linkam cell; any gases produced from Kapton decomposition would have likely been flushed out. However, this does not necessarily mean the decomposing Kapton had no effect on the samples. Studies on Kapton being heated in an N_2_ atmosphere report that CO_2_ and CO are common gaseous products, with CH_4_, H_2_, and additional N_2_ gases also forming (Lua and Su 2006). CO_2_ and N_2_ are inert, however CO is reducing and H_2_ can be oxidizing or reducing depending on other reactants present. Additionally, CH_4_ may react with other gases, whether it is water being produced by the heated Murchison samples or the other gases generated by the decomposing Kapton. These reactions include CH_4_ and water forming H_2_ and CO_2_ or CO, as well as CH_4_ and CO_2_ forming CO and H_2_ (Gong et al. 2014). Given that CO_2_ and CO are the most abundant products of Kapton decomposition (Lua and Su 2006), the main effect of Kapton decomposition would be a reducing environment within the Linkam cell. Given that post-hydration heating occurs under reducing conditions on C-complex asteroids (Nakamura 2005), the Kapton potentially producing a reducing environment was unlikely to have made the redox conditions less analogous to such processes.

### Overall mineral reactions

The mineral reactions observed are consistent with the Murchison samples being heated in an inert to reducing environment. Troilite, a mineral that cannot form under oxidizing conditions (Li et al. 2023) but can be readily produced in tochilinite-bearing CM chondrites heated in an inert atmosphere (Fuchs et al. 1973; Jenkins et al. 2024), was observed forming at 400 °C. The highest temperature at which all serpentine peaks disappeared (525 °C) is the same as that observed in a previous heating experiment with a powdered sample of Murchison (Jenkins et al. 2024). Additionally, most mineral transitions observed (tochilinite and serpentine decomposition, troilite crystallization) were consistent with previous studies of post-hydration heating of CM carbonaceous chondrites (e.g., Jenkins et al. 2024; Lindgren et al. 2020; Fuchs et al. 1973; Akai 1988, 1992). The only exception here was the detection of clinopyroxene at 500 °C in M_B. In a previous heating investigation on Murchison, clinopyroxene appeared at 575 °C due to the decomposition of calcite (Jenkins et al. 2024). The appearance of clinopyroxene here indicates that the breakdown of calcite is beginning at a lower temperature. Although inconsistent with the previous study, calcite is known to have variable decomposition temperatures depending on strain, composition, grain size, and morphology (Galan et al. 2013), with its breakdown occurring anywhere between 440 and 800 °C (Wang and Thomson 1995; Lee et al. 2019; Rodriguez-Navarro et al. 2009; Karunadasa et al. 2019). Murchison itself is a shocked meteorite (Scott et al. 1992), and with shock metamorphism being a highly heterogeneous process (Stöffler et al. 2018), the detection of clinopyroxene here is likely due to some calcite in M_B having experienced more strain than that of the sample of Murchison in our previous study (Jenkins et al. 2024).

### Models of serpentine decomposition

Here, we observe the breakdown of serpentine at 400–525 °C, followed by the appearance of a transitional structure (attributed to be dehydroxylate I) at 525 °C. The breakdown of serpentine here is consistent with previous studies on the process (e.g., Jenkins et al. 2024; Lindgren et al. 2020; Fuchs et al. 1973; Akai 1988, 1992), and the temperatures of dehydroxylate I formation also match those of previous experiments on CM chondrite meteorites (Akai 1988, 1992; Jenkins et al. 2024). Serpentine decomposition has been studied in detail, both in terrestrial and extraterrestrial contexts, leading to three main models regarding how it progresses and how its products form. These three models are the Ball and Taylor (1963) model, the Brindley and Hayami (1965) model, and the MacKenzie and Meinhold (1994) model. The earliest of these models predates the terminology of dehydroxylate I and II.

In the Ball and Taylor model, ion exchange facilitates phase changes. Serpentine is divided into donor and acceptor regions, where donor regions give Mg^2+^, Fe^2+^, and Si^4+^ in exchange for H^+^ (Eq. 1). The donor regions get outgassed as water, while the acceptor regions form a partially disordered structure that then further divides into Mg^2+^, Fe^2+^-rich and Si^4+^-rich regions, which crystallize into olivine and pyroxene, respectively. Although this is the general order in which serpentine decomposition would progress, multiple stages can occur simultaneously (Ball and Taylor 1963). The composition of the partially disordered structure in Eq. 1 is not meant to be exact, but rather an approximation Ball and Taylor (1963) used for illustrative purposes, however they specified that its (Mg,Fe):Si ratio should be 3:2, in common with serpentine (After Ball and Taylor 1963).

1 $$\begin{aligned} & 7\left( {{\text{Mg}},{\text{Fe}}} \right)_{3} {\text{Si}}_{2} {\text{O}}_{9} {\text{H}}_{4} \left( {acceptor} \right) + 2\left( {{\text{Mg}},{\text{Fe}}} \right)_{3} {\text{Si}}_{2} {\text{O}}_{9} {\text{H}}_{4} \left( {donor} \right) \\ & \to \left( {Mg,Fe} \right)_{27} {\text{Si}}_{18} {\text{O}}_{63} \left( {acceptor} \right) + 18{\text{H}}_{2} {\text{O}} \left( {donor} \right) \\ \end{aligned}$$

The Brindley and Hayami model builds on the Ball and Taylor model, with ion exchange between acceptor and donor regions being the mechanism for the transformation of chrysotile and serpentine into anhydrous silicates. However, Brindley and Hayami (1965) argue that because olivine is the main product of this process, less Si^4+^ is transferred to acceptor regions from donor regions, with donor regions becoming a mixture of water (which also gets outgassed) and SiO_2_ (Eq. 2). The acceptor region crystallizes exclusively into olivine, with pyroxene only forming as a reaction between SiO_2_ and olivine at higher temperatures (After Brindley and Hayami 1965).

2 $$\begin{aligned} & 4\left( {{\text{Mg}},{\text{Fe}}} \right)_{3} {\text{Si}}_{2} {\text{O}}_{9} {\text{H}}_{4} \left( {acceptor} \right) + 2\left( {{\text{Mg}},{\text{Fe}}} \right)_{3} {\text{Si}}_{2} {\text{O}}_{9} {\text{H}}_{4} \left( {donor} \right) \\ & \to 9\left( {{\text{Mg}},{\text{Fe}}} \right)_{2} {\text{SiO}}_{4} \left( {acceptor} \right) + 12{\text{H}}_{2} {\text{O}} + 3{\text{SiO}}_{2} \left( {donor} \right) \\ \end{aligned}$$

The most recent model is the MacKenzie and Meinhold model, wherein Mg^2+^, Fe^2+^-rich and Si^4+^-rich regions form through cation exchange synchronously with the outgassing of water. The Mg^2+^, Fe^2+^-rich regions produce dehydroxylate I, which is followed by the formation of dehydroxylate II from the Si^4+^-rich regions (Eq. 3). Dehydroxylate I recrystallizes into olivine first, while dehydroxylate II eventually forms a mixture of pyroxene and SiO_2_. The SiO_2_ from dehydroxylate II then reacts with olivine to form more pyroxene, similar to that in the preceding Brindley and Hayami model (After MacKenzie and Meinhold 1994):

3 $$\begin{aligned} & 5\left( {{\text{Mg}},{\text{Fe}}} \right)_{3} {\text{Si}}_{2} {\text{O}}_{9} {\text{H}}_{4} \to 7\left( {{\text{Mg}},{\text{Fe}}} \right)_{2} {\text{SiO}}_{4} \left( {dehydoxylate I} \right) \\ & \quad + \left( {{\text{Mg}},{\text{Fe}}} \right)_{3} {\text{SiO}}_{7} \left( {dehydoxylate II} \right) + 10{\text{H}}_{2} {\text{O}} \\ \end{aligned}$$

### Dehydroxylate I identification and formation

The 3.51 Å peak associated with dehydroxylate I was only identified in a handful of targets in each sample despite the abundance of serpentine in Murchison (~ 20–70 vol.%, Howard et al. 2009). The few targets identified are likely due to a combination of sample shifting throughout the experiment, sample heterogeneity, preferred orientation, and dehydroxylate I formation proceeding heterogeneously. Here, samples M_A and M_B moved around the collection area as Kapton melted which, in the case of M_A, significantly reduced the data collection area, leading to a smaller number of serpentine targets. Murchison is a CM chondrite meteorite composed of a plethora of mineral phases (Fig. 4) that make up large chondrules and a polymineralic matrix; large groupings of serpentine necessary for dehydroxylate I formation are not evenly spaced. As μXRD lacks the powder averaging of multiple diffracting domains that pXRD has, preferred orientation plays a greater role. Here, we relied on the 3.5 Å to identify dehydroxylate I, however if dehydroxylate I was not in the right orientation to give this particular peak, it would not have been detected. Finally, it is unlikely that the serpentine to dehydroxylate I transition proceeds homogeneously, and dehydroxylate II areas would also form in the process. It may be possible that if held at temperature for longer, more targets showing the 3.5 Å peak may have been observed.

**Fig. 7:**
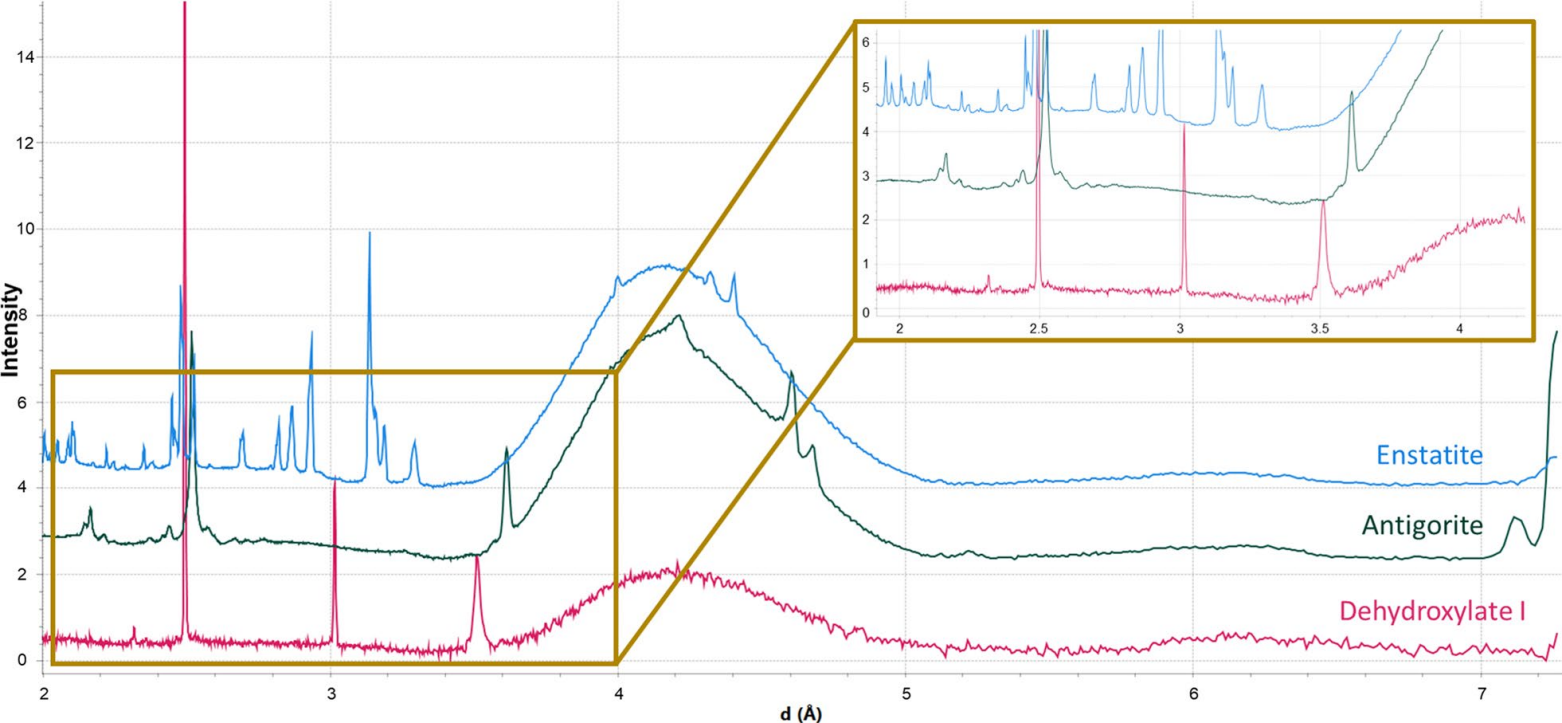
Dehydroxylate I in M_B at 525 °C (pink) compared to the enstatite (blue) and antigorite (green). Enstatite had some peak overlap with dehydroxylate I in a previous study (Jenkins et al. 2024), while antigorite is a serpentine-family mineral related to carlosturanite. Dehydroxylate I matches neither standard

The d-spacings we observed for dehydroxylate I did not match any of the standards used. However, some of the peaks are likely to be modified serpentine peaks, with some peaks appearing to result from shifting serpentine peaks (e.g., 3.51 Å), while the 2.49 Å lines up with the lizardite standard (Fig. 6) despite the lack of other serpentine peaks throughout the sample. Peak positions of the d-spacings did not match enstatite (Fig. 7), which overlapped with some dehydroxylate I peaks in a previous investigation we conducted (Jenkins et al. 2024). Dehydroxylate I’s d-spacings were further compared to minerals in the COD. No matches were found. Further investigation into various databases (e.g., American Mineralogist Crystal Structure Database; RRUFF) and papers revealed that the d-spacings only matched that of the mineral carlosturanite ((Mg,Fe)_21_Si_12_O_28_(OH)_34_·H_2_O), whose XRD pattern was first characterized by Compagnoni et al. (1985) (Table 1).

**Table 1:** Peaks attributed to dehydroxylate I compared to carlosturanite peaks

Dehydroxylate I	Carlosturanite^a^	
d-spacing (Å)	d-spacing (Å)	hkl
3.67^b^	3.65	$\overline{2 }$ 02
3.56^b^	3.58	002
3.51/3.54^b^	3.52	801
3.04	3.00	12·00
2.54	2.57	802
2.49	2.55	$\overline{12 }$·02
2.46	2.43	$\overline{2 }$ 03
2.35	2.31	203
2.31	2.30	$\overline{8 }$ 03

^a^Not a complete list of carlosturanite peaks. For all carlosturanite peaks, see Compagnoni et al.(1985)

^b^Observed by Jenkins et al. (2024) and attributed to dehydroxylate I.

Carlosturanite is a mineral related to antigorite that occurs in serpentinites (Compagnoni et al. 1985). Here, dehydroxylate I did not match antigorite (Fig. 7), but given dehydroxylate I’s comparable d-spacings with carlosturanite (Table 1) and with antigorite being a serpentine-group mineral, carlosturanite seems like a perfect match. Yet, there is one key aspect regarding carlosturanite that negates this match: carlosturanite decomposes at 400 °C (Compagnoni et al. 1985), while dehydroxylate I is observed forming at 525 °C. However, X-ray diffraction is the study of crystal structure, and minerals with similar structures will also have similar diffraction patterns. A classic example of this is the spinel group minerals (e.g., magnetite, hematite, magnesioferrite) often having highly similar diffraction patterns. Dehydroxylate I is not carlosturanite, but rather it may have a crystal structure that shares some similarities to it.

**Fig. 8:**
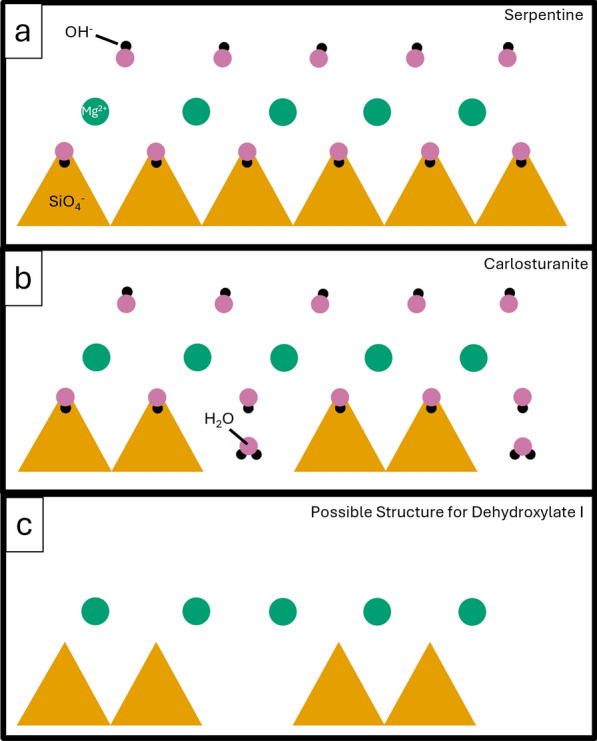
Diagrams of crystal structures of serpentine, carlosturanite, and dehydroxylate I. Diagrams are drawn perpendicular to the c-axis and flattened for illustrative purposes. For simplicity only the Mg-rich endmembers (e.g., lizardite) are represented here. SiO_4_^−^ tetrahedra are represented by yellow triangles. Green circles represent Mg^2+^, while black circles represent H^+^ and pink circles represent O^2−^. a) Diagram of serpentine showing a continuous tetrahedral (SiO_4_^−^) and octahedral (Mg^2+^) sheets with OH^−^ present. b) Diagram of carlosturanite with a continuous octahedral (Mg^2+^) sheet and a discontinuous tetrahedral (SiO_4_^−^) sheet. Breaks in the tetrahedral sheet are infilled with H_2_O. Like serpentine, OH^−^ is also present. For a more detailed view of the carlosturanite crystal structure, please see Mellini et al. (1985). c) Diagram of the possible structure of dehydroxylate I. The octahedral (Mg^2+^) sheet is continuous, but the tetrahedral (SiO_4_^−^) sheet has gaps. H_2_O and OH^−^ are absent, with OH^−^ having been gradually outgassed in heating

Carlosturanite could be used as a starting point to understand the structure of dehydroxylate I. Like serpentine and antigorite, carlosturanite is composed of an octahedral sheet (e.g., Mg^2+^, Fe^2+^) and a tetrahedral sheet (SiO_4_^−^) with OH^−^ between layers (Mellini et al. 1985). Unlike serpentine and antigorite, carlosturanite has less Si^4+^ and its tetrahedral sheet is broken into triple chains connected by H_2_O (Mellini et al. 1985). We propose that dehydroxylate I may have a similar structure to carlosturanite, having an octahedral sheet and a tetrahedral sheet broken up into triple chains, but differs in that the OH^−^ and H_2_O are likely absent, with OH^−^ degassed during heating and H_2_O never being present in the first place (Fig. 8).

This proposed structure for dehydroxylate I would be consistent with how Mg^2+^, Fe^2+^-rich regions form in serpentine decomposition models by both Ball and Taylor (1963) and MacKenzie and Meinhold (1994), wherein these regions are formed through the removal of Si^4+^. Additionally, both Ball and Taylor (1963) and MacKenzie and Meinhold (1994) reported that the heating of chrysotile and serpentine under dry conditions, like those in this study, leads to the tetrahedral layer to be significantly less stable than that of the octahedral sheet. This is also consistent with previous work by Zulumyan et al. (2014; 2018), wherein through acid-leaching studies of heated serpentine they found that siloxane bonds in the tetrahedral sheet readily broke down during heating. The removal of Si^4+^ from serpentine’s crystal structure may allow the gaps in the tetrahedral sheet required to form triple chains similar to that of carlosturanite.

The various models for serpentine decomposition do not require serpentine to become completely amorphous during decomposition. This is consistent with Jenkins et al. (2024), where in ALH 83100 secondary olivine started forming at ~ 575–600 °C prior to complete decomposition of serpentine at 700 °C. Dehydroxylate I has been found to occur at 400–600 °C in heated meteorites (Akai 1988, 1992; Jenkins et al. 2024); the identification of dehydroxylate I in naturally heated meteorites would narrow the range of possible peak temperatures experienced by these meteorites to 400–600 °C, while the abundance of other related phases (e.g., serpentine, olivine) may have implications regarding timescale. Dehydroxylate I is a transitional phase between serpentine decomposition and olivine crystallization; if peak heating temperature is known, the relative abundances of these three phases can be used to infer timescale. Olivine begins to form at ≥ 500 °C (Akai 1992; Ball and Taylor 1963), and thus it is predicted that if a meteorite is heated for a long enough duration at those temperatures, any dehydroxylate I formed would completely recrystallize into olivine. The proportion of dehydroxylate I to olivine could be related to the timescale of post-hydration heating at 500–600 °C, with more olivine being indicative of longer heating durations. Likewise, the proportion of crystalline serpentine to dehydroxylate I could lead to similar inferences, as lower dehydroxylate I abundances relative to crystalline serpentine would be evidence of very short heating timescales (e.g., hours).

A pXRD pattern for dehydroxylate I has yet to be produced, however the XRD peaks occurring at the d-spacings identified here (Table 1) can be used to determine its presence in moderately heated CM chondrite meteorites. This in turn will aid in evaluating the temperature-timescales experienced by heated CM chondrite meteorites, which will have implications regarding the processes experienced by the asteroidal volatile sources within our early solar system.

## Conclusions

Dehydroxylate I formed by serpentine decomposition in CM chondrites has been successfully isolated at 525 °C using µXRD. Its µXRD pattern matches the antigorite-related mineral carlosturanite, but the two phases are not the same as carlosturanite decomposes at 400 °C. Nonetheless, dehydroxylate I may have similarities in its crystal structure to carlosturanite, developing as gaps in serpentine’s tetrahedral sheet as Si^4+^ is removed during decomposition, which is consistent with both the Ball & Taylor (1963) and the MacKenzie & Meinhold (1994) models for serpentine decomposition. We have identified the d-spacings at which dehydroxylate I’s XRD peaks occur, which can be used to help interpret XRD patterns of naturally and experimentally heated serpentine-bearing samples including CM chondrite meteorites. By constraining the temperature-timescales of the mineral reactions experienced by these meteorites due to heating, a better understanding of volatile sources within the early solar system will be achieved.

## Data Availability

The data collected and analysed for this study are available in the Zenodo repository at 10.5281/zenodo.15221889. These datasets are composed of .nxs files and require Data Analysis WorkbeNch (DAWN) software to view. Use the DataVis perspective in DAWN with the plot type set to Hyper3d to view the data.

## Abbreviations

ALH 83100: Allan Hills 83100
CM chondrite: ‘Mighei-like’ carbonaceous chondrite
DAWN: Data Analysis WorkbeNch
DLS: Diamond Light Source
pXRD: Powdered X-ray diffraction
μXRD: Micro X-ray diffraction
